# Abnormal Cardiac PET Imaging in ATTR Amyloidosis

**DOI:** 10.1016/j.jaccas.2026.108678

**Published:** 2026-09-23

**Authors:** Mihir Shah, Jacob Agronin, Jared Hassler, Glenn Gerhard, Sanjana Kashinath, David Laslett, Carly Fabrizio

**Affiliations:** a Department of Internal Medicine, Temple University Hospital, Philadelphia, Pennsylvania, USA; b Department of Cardiology, Temple University Hospital, Philadelphia, Pennsylvania, USA; c Department of Pathology, Temple University Hospital, Philadelphia, Pennsylvania, USA; d Department of Medical Genetics and Molecular Biochemistry, Lewis Katz School of Medicine at Temple University, Philadelphia, Pennsylvania, USA; e Department of Hematology, Temple University Hospital, Philadelphia, Pennsylvania, USA; f Department of Cardiology, Inova, Fairfax, Virginia, USA

**Keywords:** cardiomyopathy, imaging, nuclear medicine, positron emission tomography

## Abstract

**Background:**

Transthyretin amyloidosis (ATTR) is an infiltrative cardiomyopathy caused by deposition of misfolded transthyretin protein in the myocardium. Diagnosis typically relies on technetium-99m–based imaging or magnetic resonance imaging.

**Case Summary:**

A 72-year-old man presented for evaluation of multifocal premature ventricular contractions and nonsustained ventricular tachycardia after atrial fibrillation ablation. Cardiac positron emission tomography (PET) was highly suggestive of cardiac sarcoidosis. However, voltage-guided endomyocardial biopsy revealed a tissue diagnosis of ATTR.

**Discussion:**

Although PET typically identifies inflammatory disease, this case suggests that fluorodeoxyglucose uptake may also occur in ATTR.

**Take-Home Messages:**

Diagnosis of ATTR often requires multimodality imaging to identify and differentiate from other infiltrative cardiomyopathies. Cardiac PET, although not classically used for this application, may have utility in the diagnostic pathway.

## Visual Summary

**Figure:**
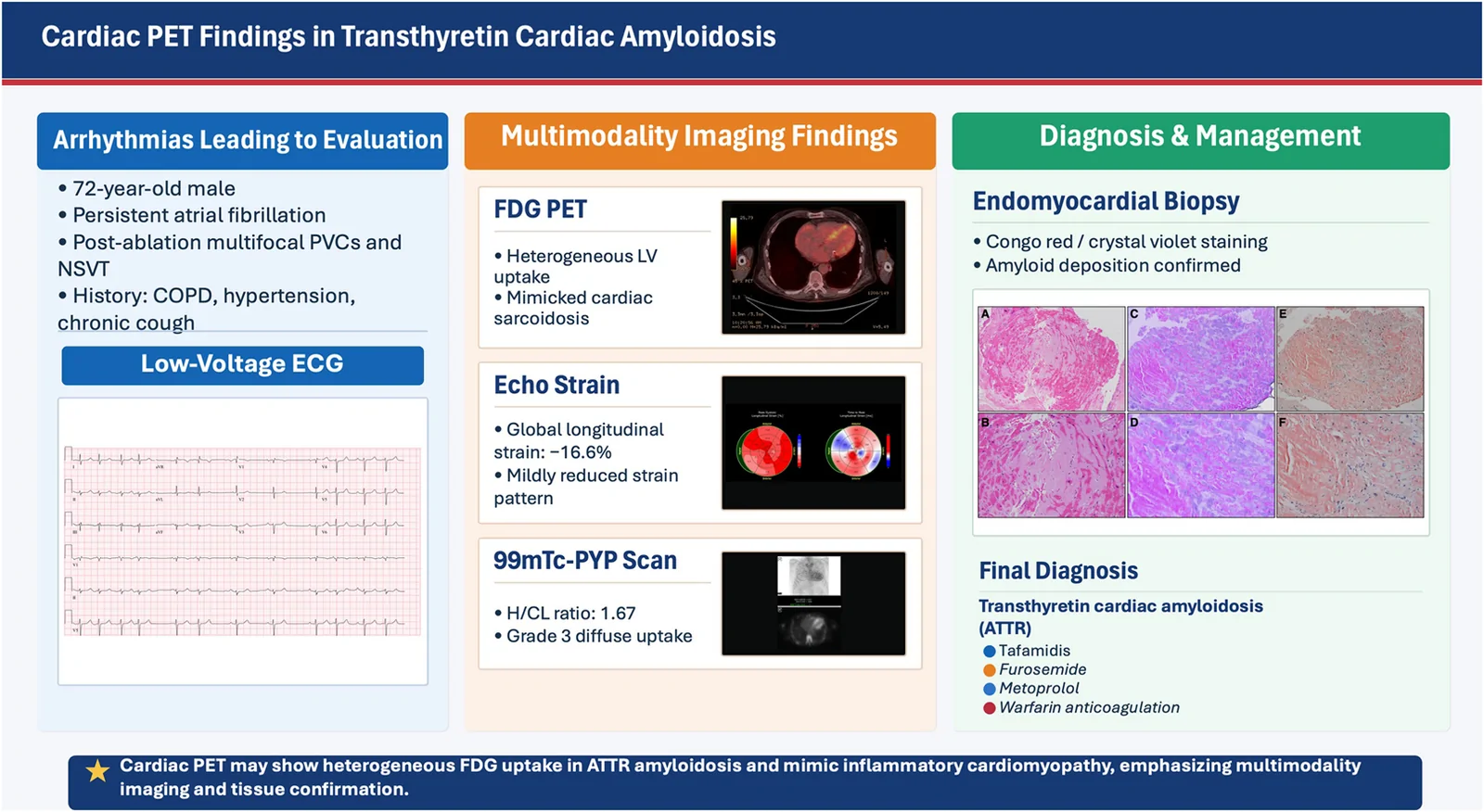

## History of Presentation

A 72-year-old man with shortness of breath in the context of newly diagnosed persistent atrial fibrillation presented for outpatient electrophysiology evaluation. He developed recurrence of atrial fibrillation despite cardioversion and underwent atrial fibrillation ablation. Ambulatory monitoring post-ablation revealed multifocal premature ventricular contractions (PVC) burden and nonsustained ventricular tachycardia (NSVT).

## Past Medical History

The patient had a past medical history of mild chronic obstructive pulmonary disease (COPD), chronic cough, hypertension, and persistent atrial fibrillation.

## Differential Diagnosis

The possible differential diagnosis for coexisting atrial arrhythmias and multifocal PVCs/NSVT includes infiltrative cardiac disease such as cardiac sarcoid, cardiac amyloid, and hemochromatosis, as well as an alternative nonischemic cardiomyopathy without manifest left ventricular (LV) dysfunction, including myocarditis and hereditary causes that may first manifest with arrhythmias (such as mutations in lamin A/C, desmosomal proteins, and some sarcomeric proteins).

## Investigations

**Figure 1:**
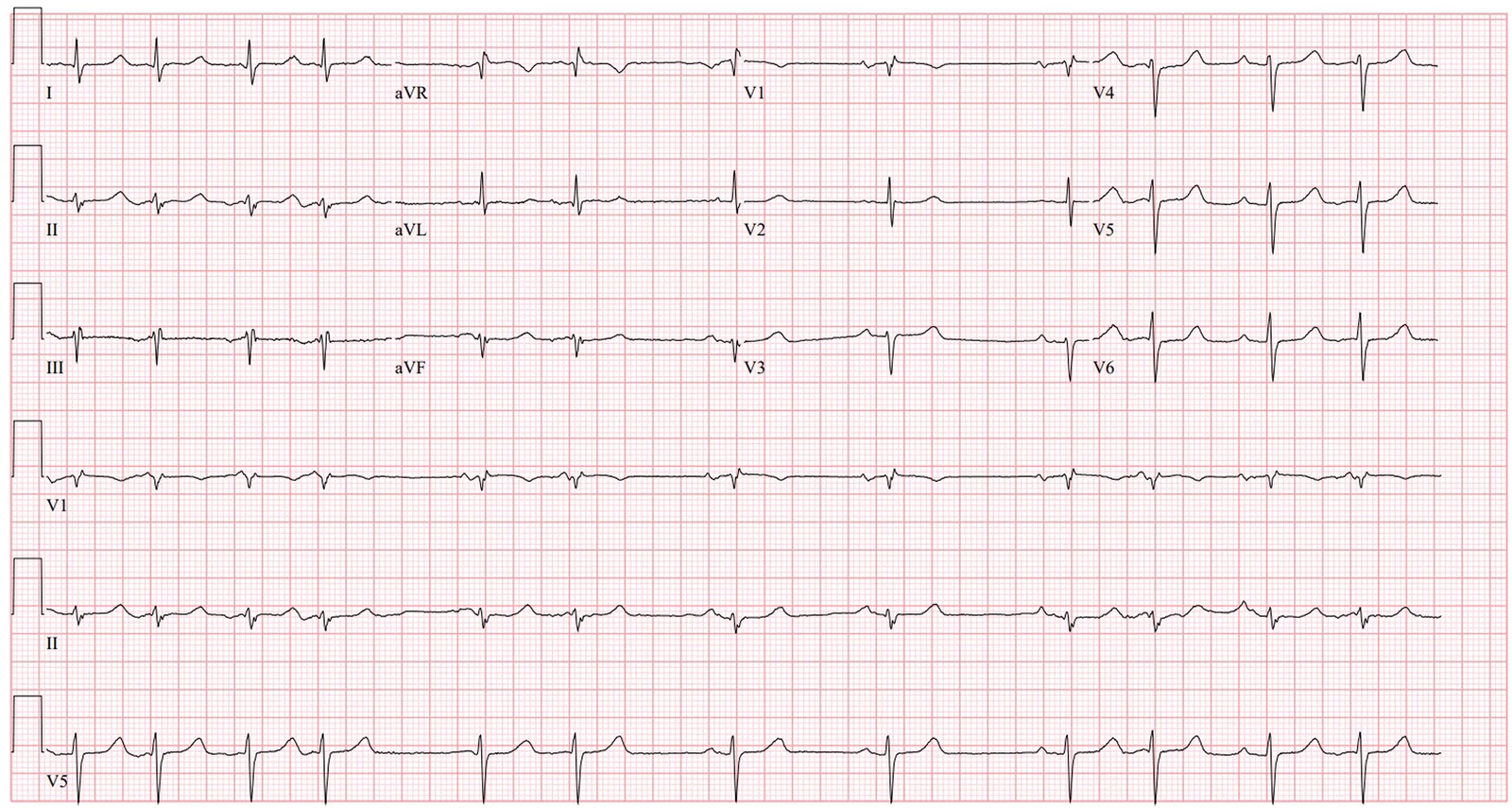
Electrocardiogram Electrocardiogram demonstrates sinus rhythm with frequent atrial ectopy with inappropriately normal voltage.

Initial echocardiography revealed low normal LV systolic function (ejection fraction of 50%) with an upper normal LV wall thickness of 11 mm, mildly increased LV mass index, and moderately dilated left and right atria (Video 1). Electrocardiogram obtained after atrial fibrillation ablation showed inappropriately normal voltage in the limb leads despite increased LV mass index (Figure 1). Computed tomography obtained for COPD demonstrated calcified granulomas and mediastinal lymphadenopathy.

**Figure 2:**
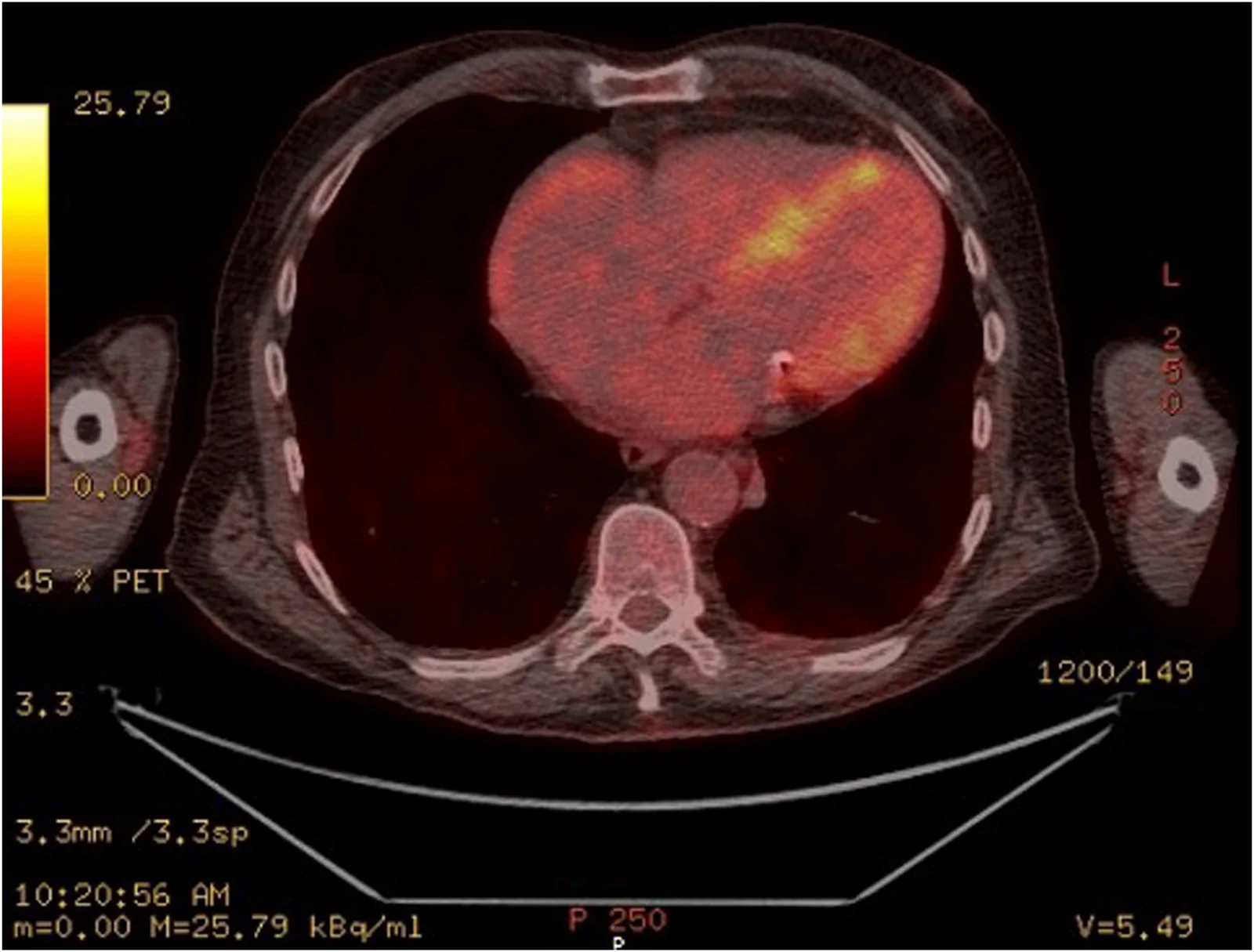
Cardiac PET/Computed Tomography Positron emission tomography (PET) scan shows heterogeneous fluorodeoxyglucose uptake in the left ventricular myocardium.

The patient underwent ablation for atrial fibrillation, and subsequent Zio (iRhythm) monitoring showed 3% multivocal PVCs and 18 episodes of NSVT, the longest run lasting 16.3 seconds. Cardiac positron emission tomography (PET) was ordered because of suspicion for cardiac sarcoid, which showed diffuse heterogeneous fluorodeoxyglucose (FDG) uptake in the LV myocardium and bilateral atrial wall and hypermetabolic mediastinal lymph nodes (Figure 2). Blood glucose at time of PET was 74 mg/dL. Cardiac magnetic resonance (CMR) was deferred owing to severe claustrophobia.

**Figure 3:**
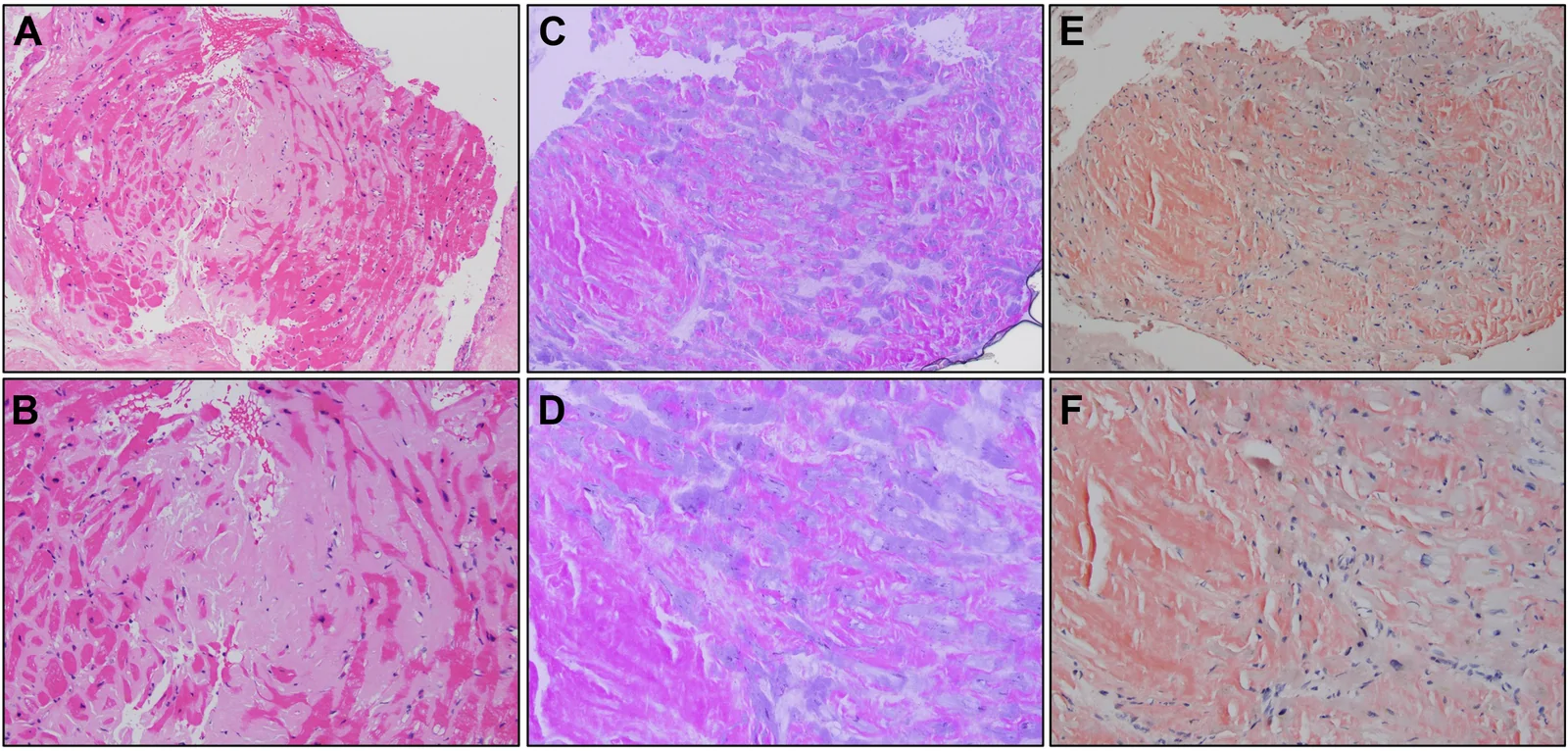
Histopathology of Myocardial Endomyocardial Biopsy Samples (A and B) Hematoxylin and eosin stain shows an amorphous, acellular material filling within the extracellular space. (C and D) Crystal violet stain highlights fuchsinophilic fibrils. (E and F) Congo red stain highlights the amorphous material.

Mediastinal lymph node biopsy specimens showed lymphocytes and bronchial tissue, but were negative for granulomas and malignancy; specimens were never stained for amyloid. These findings prompted further evaluation with voltage gated endomyocardial biopsy (EMB). Samples obtained from the right ventricular septum showed positive Congo red and crystal violet stains consistent with cardiac amyloidosis (Figures 3A to 3F), with no evidence of cardiac sarcoid. Serum immunoglobulin assessment demonstrated mild polyclonal gammopathy and normal serum free light chains when adjusted for glomerular filtration rate.

**Figure 4:**
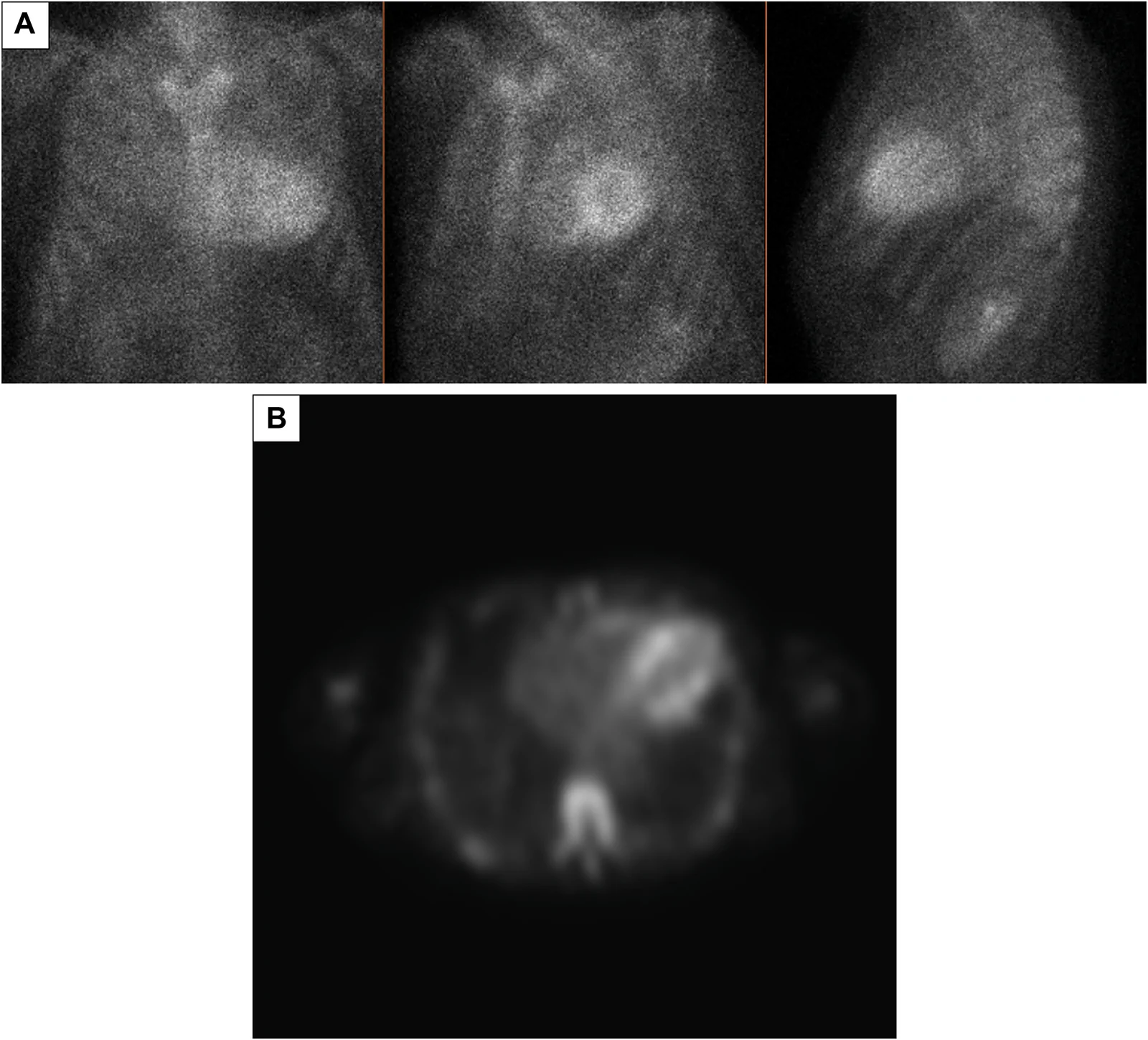
Technetium 99m Pyrophosphate Imaging (A) Diffuse myocardial uptake on anterior, left anterior oblique, and lateral views. (B) Single-photon emission computed tomography image showing diffuse myocardial uptake isolated from blood pool.

**Figure 5:**
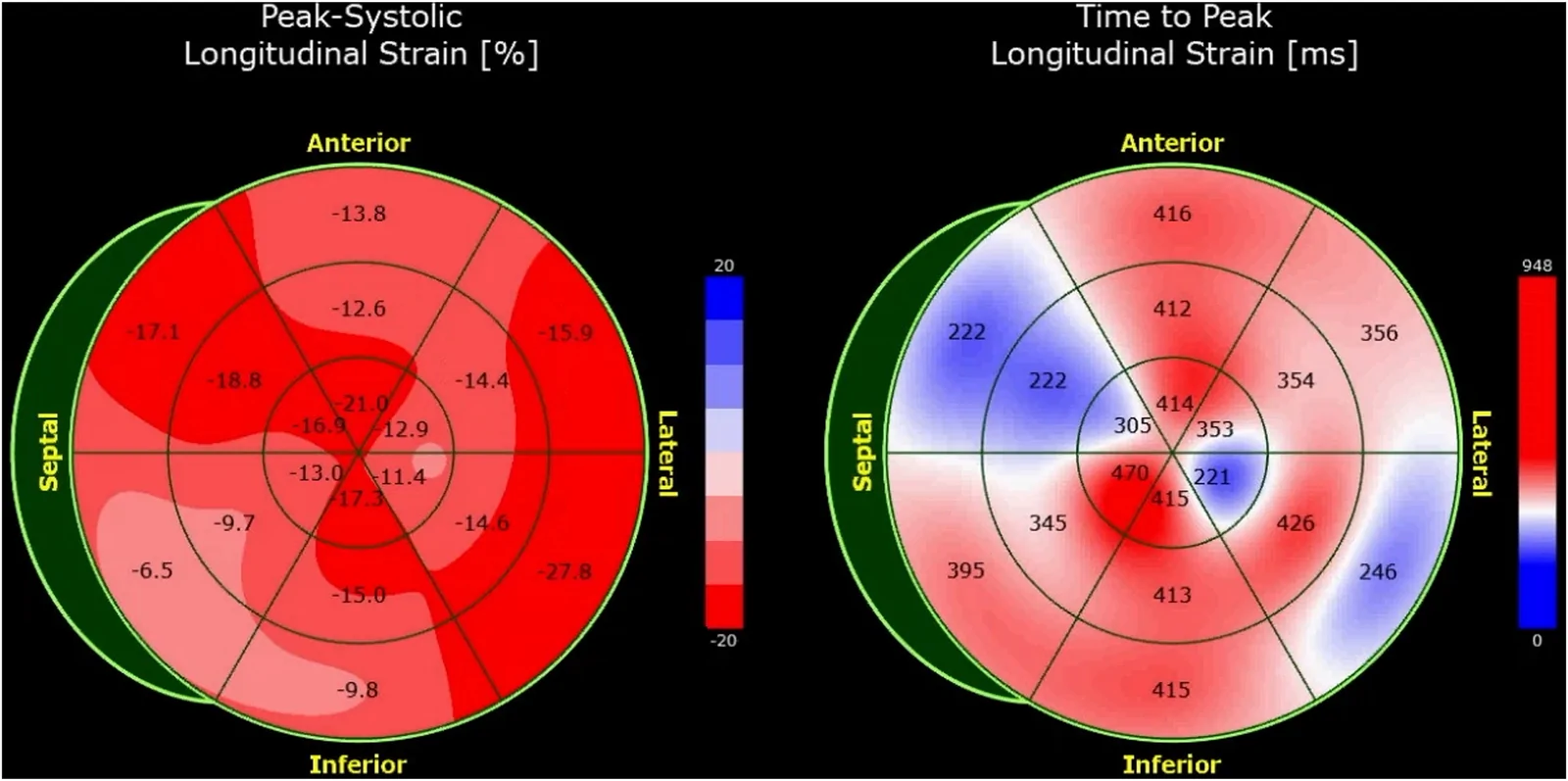
Echocardiogram With Strain Mildly reduced global longitudinal strain of −16.6%.

Technetium 99m pyrophosphate (^99m^Tc-PYP) scan showed diffuse myocardial uptake on anterior, left anterior oblique, and lateral views with single-photon emission computed tomography showing uptake isolated from blood pool, which is highly suggestive of transthyretin amyloidosis (ATTR) (Figures 4A and 4B). Follow-up transthoracic echocardiography with strain showed mildly reduced global longitudinal strain of −16.6% (Figure 5), though with significant heart rate variation. Right ventricular size was upper limit of normal with mildly reduced systolic function. Arrhythmia limited assessment of diastolic function. Mass spectroscopy of the EMB sample revealed peptides consistent with TTR deposition without TTR abnormalities, further corroborated by the absence of TTR variants on the Invitae Arrhythmia and Cardiomyopathy Comprehensive Panel (Labcorp). Genetic testing, however, did reveal a known pathogenic desmin variant, and variants in this sarcomeric protein can manifest with arrhythmias before a dilated cardiomyopathy.^1^ B-type natriuretic peptide was 439.1 pg/mL, and high-sensitivity troponin was 61 ng/L.

## Management

The patient was initially started on inhaled long-acting muscarinic antagonist therapy for mild COPD with minimal improvement in dyspnea. The patient underwent an elective catheter ablation for atrial fibrillation with improvement in symptoms after direct current cardioversion failed. After recurrence of persistent atrial fibrillation 7 months later, transesophageal echocardiography before repeat direct current cardioversion and amiodarone initiation revealed left atrial appendage thrombus, which is known to occur more frequently in cardiac amyloid, and cardioversion had to be aborted.^2^ The patient was switched from apixaban to warfarin, and adequate rate control was achieved with atrioventricular nodal blockade. The patient also developed new clinical heart failure and was started on daily furosemide with significant improvement in symptoms. After confirmation of the diagnosis of ATTR, tafamidis was started.

## Outcome

The patient was maintained on daily furosemide, metoprolol, and tafamidis. Pending resolution of left atrial appendage thrombus, rhythm control for atrial fibrillation after switching to warfarin is planned. The patient is undergoing evaluation with a urologist including magnetic resonance imaging and potential prostate biopsy with Congo red stain. The Invitae genetic panel revealed an incidental pathogenic variant in desmin, which may have contributed to his ventricular arrhythmias in addition to ATTR.

## Discussion

Cardiac amyloidosis (CA) is a progressive disease characterized by deposition of misfolded amyloid proteins within the myocardium. The 2 predominant forms of CA are ATTR and immunoglobulin light chain amyloidosis. ATTR is caused by either hereditary mutation in the transthyretin gene (variant) or misfolding of protein with age (wild-type), whereas immunoglobulin light chain amyloidosis is a disease of monoclonal plasma cell proliferation. CA carries significant mortality when symptoms of heart failure develop, with a median survival for ATTR of 3 to 5 years.^3^

Once the diagnosis of CA is suspected, current consensus guidelines recommend screening with ^99m^Tc-PYP imaging and assessment of serum immunoglobulins. Endomyocardial biopsy should be considered if ^99m^Tc-PYP is unavailable or suggestive of CA. Definitive diagnosis of CA is made by positive Congo red staining of EMB and mass spectroscopy, which should be followed by genetic testing for mutations in the transthyretin protein to distinguish between wild-type and variant ATTR.^4^ CMR is another established imaging modality used in the diagnosis of CA, characterized typically by diffuse subendocardial or transmural late gadolinium enhancement in the myocardium or an inability to null the myocardium during image acquisition.^5^

Cardiac PET is not routinely recommended in the diagnosis of CA as the tracer selectively highlights inflammatory tissue. Regarding the pathogenic desmin mutation, the literature also does not include any studies evaluating desmin cardiomyopathy with PET, as CMR is the primary imaging modality. The mechanism for FDG uptake in this classically noninflammatory condition is currently unknown. However, studies have shown the utility of PET with FDG in distinguishing local deposition from systemic amyloidosis without specific organ involvement.^6^ Some studies suggest that an inflammatory or cleanup response by macrophages, monocytes, and giant cells may contribute to PET positivity and may be present in a heterogeneous pattern in the tissue.^7^ Several studies show how major immune cell types are detectable in ATTR stain and investigate the role of inflammation in cardiac amyloid. These studies describe how 3 major immune cell types (T lymphocytes, macrophages, and neutrophils) are present in various capacities in ATTR cardiac tissue. Although there is no consensus on a correlation between these cell types and the clinical course of amyloid, worse clinical outcomes occur in the presence of inflammation, suggesting a pathophysiological role in the disease.^8^^,^^9^ Other limited reports in the literature suggest the possibility of false-positive PET results, which is less likely in this case owing to proper preparation and blood glucose within acceptable range.^10^ In this case, the decision to proceed with EMB was based on high suspicion of infiltrative disease on cardiac PET, suggesting a role for this imaging modality in the diagnosis of CA.

## Conclusions

Cardiac PET imaging can show heterogeneous FDG uptake in ATTR with unclear prognostic or therapeutic significance. Further research, including different PET modalities, is needed. ATTR may manifest with atrial and ventricular arrhythmias before the onset of heart failure or significant LV hypertrophy.

**Visual Summary:**
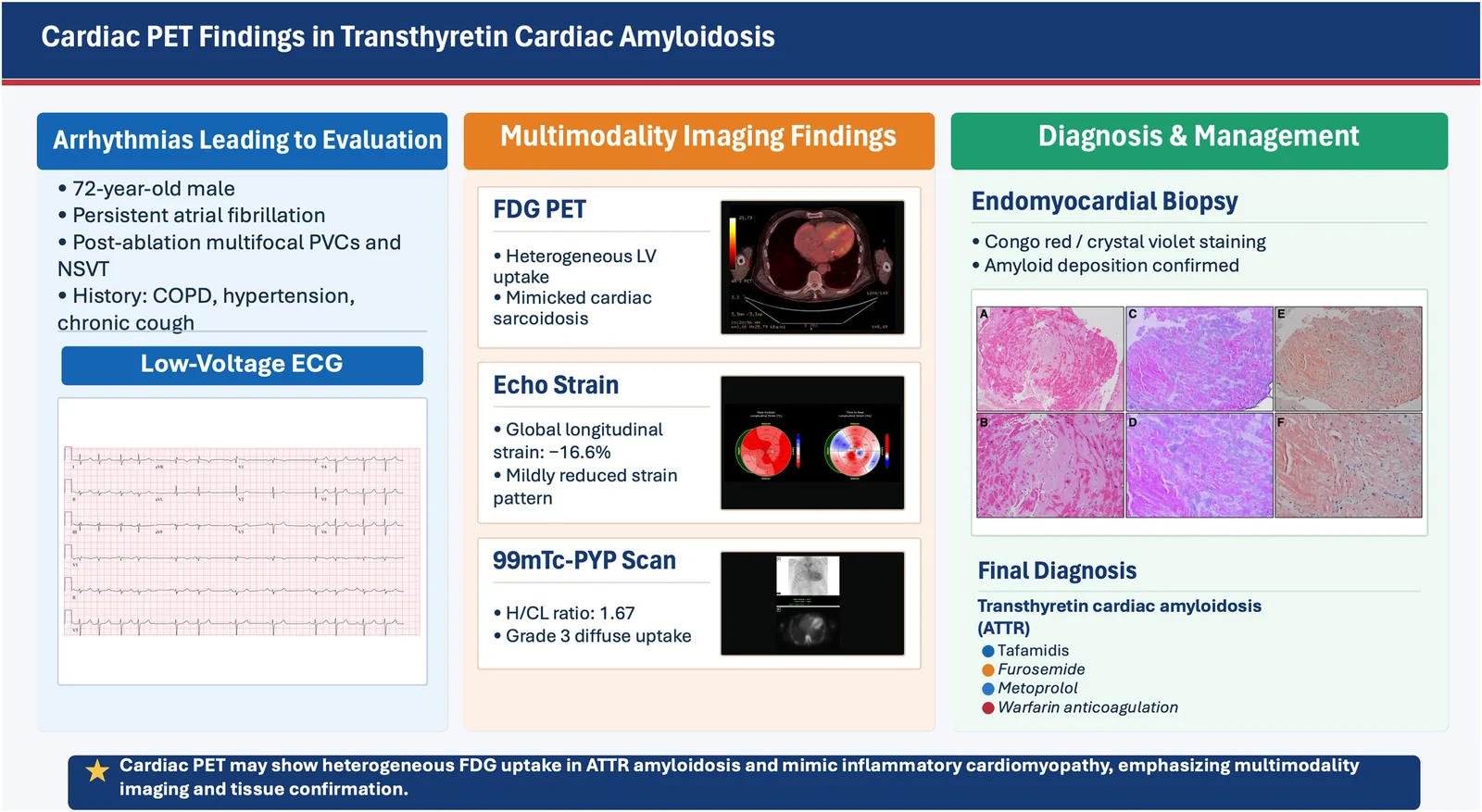
Clinical Course With Presenting Symptoms, Imaging Work-Up, Biopsy Findings, and Management ATTR = transthyretin amyloidosis; COPD = chronic obstructive pulmonary disease; ECG = electrocardiogram; FDG = fluorodeoxyglucose; LV = left ventricular; NSVT = nonsustained ventricular tachycardia; PVC = premature ventricular contraction; ^99m^Tc-PYP = technetium 99m pyrophosphate.

## Funding Support and Author Disclosures

The authors have reported that they have no relationships relevant to the contents of this paper to disclose.

**Take-Home Messages**

• Diagnosis of ATTR often requires multimodality imaging to identify and differentiate this cardiomyopathy from other infiltrative cardiomyopathies.
• Cardiac PET, although not classically used for this application, may have utility in the diagnostic pathway.

## Appendix

Video 1 Echocardiogram Parasternal long axis view with increased interventricular septum diameter and mildly reduced ejection fraction.
